# A Novel Lipase from *Streptomyces exfoliatus* DSMZ 41693 for Biotechnological Applications

**DOI:** 10.3390/ijms242317071

**Published:** 2023-12-02

**Authors:** Guillermo Rodríguez-Alonso, Juan Toledo-Marcos, Lara Serrano-Aguirre, Carlos Rumayor, Beatriz Pasero, Aida Flores, Ana Saborido, Pilar Hoyos, María J. Hernáiz, Isabel de la Mata, Miguel Arroyo

**Affiliations:** 1 Department of Biochemistry and Molecular Biology, Faculty of Biology, Universidad Complutense de Madrid (UCM), E-28040 Madrid, Spain; guillr05@ucm.es (G.R.-A.); juan.toledo-marcos@medunigraz.at (J.T.-M.); lara.serrano@cib.csic.es (L.S.-A.); carlorum@ucm.es (C.R.); beatriz.pasero@cib.csic.es (B.P.); asaborid@ucm.es (A.S.); 2 Department of Chemistry in Pharmaceutical Sciences, Faculty of Pharmacy, Universidad Complutense de Madrid (UCM), E-28040 Madrid, Spain; aiflores@ucm.es (A.F.); phoyosvi@ucm.es (P.H.); mjhernai@ucm.es (M.J.H.)

**Keywords:** lipase, *Streptomyces exfoliatus*, sugar fatty acid esters, polyester biodegradation

## Abstract

Genome mining of *Streptomyces exfoliatus* DSMZ 41693 has allowed us to identify four different lipase-encoding sequences, and one of them (*Se*LipC) has been successfully cloned and extracellularly expressed using *Rhodococcus* sp. T104 as a host. *Se*LipC was purified by one-step hydrophobic interaction chromatography. The enzyme is a monomeric protein of 27.6 kDa, which belongs to subfamily I.7 of lipolytic enzymes according to its phylogenetic analysis and biochemical characterization. The purified enzyme shows the highest activity at 60 °C and an optimum pH of 8.5, whereas thermal stability is significantly improved when protein concentration is increased, as confirmed by thermal deactivation kinetics, circular dichroism, and differential scanning calorimetry. Enzyme hydrolytic activity using *p*-nitrophenyl palmitate (pNPP) as substrate can be modulated by different water-miscible organic cosolvents, detergents, and metal ions. Likewise, kinetic parameters for pNPP are: *K_M_* = 49.6 µM, *k_cat_* = 57 s^−1^, and *k_cat_/K_M_* = 1.15 × 10^6^ s^−1^·M^−1^. *Se*LipC is also able to hydrolyze olive oil and degrade several polyester-type polymers such as poly(butylene succinate) (PBS), poly(butylene succinate)-*co*-(butylene adipate) (PBSA), and poly(ε-caprolactone) (PCL). Moreover, *Se*LipC can catalyze the synthesis of different sugar fatty acid esters by transesterification using vinyl laurate as an acyl donor, demonstrating its interest in different biotechnological applications.

## 1. Introduction

Lipases (triacylglycerol hydrolases, EC 3.1.1.3) are ubiquitous enzymes that catalyze the hydrolysis of triglycerides into glycerol and fatty acids at the lipid–water interface, as well as esterification, interesterification, transesterification, acidolysis, and aminolysis reactions in aqueous and organic media [1]. These enzymatic reactions offer numerous advantages over traditional chemical methods, including higher product quality and lower manufacturing costs, as well as less waste and reduced energy consumption [2]. Consequently, lipases are used in numerous and versatile industrial and biotechnological applications, covering different areas such as the production of detergents, food, polymers, biofuels, and fine chemicals, as well as clinical diagnosis and bioremediation [3]. Among all the different lipases described, those from microbial sources (bacteria, yeasts, and fungi) have received much attention in the industry due to their properties and advantages [4], and therefore, their economic impact has expanded. According to The Brainy Insights [5], a company that provides global and regional market research reports, the microbial lipase market value worldwide was estimated at USD 518.40 million in 2021, and it is expected to reach USD 898.40 million by 2030 (a CAGR of 6.30% from 2022 to 2030).

In this context, microbial lipases have proven to solve a challenge in sustainable applied biocatalysis: the regioselective synthesis of glycosylated lipids [6]. Glycosylated lipids (GLs), such as sugar fatty acid esters (SFAEs), are considered non-ionic biosurfactants with promising applications in the food, pharmaceutical, and personal care industries [7] due to their emulsifying and biological properties [8,9]. In addition, SFAEs are biodegradable, non-toxic, and non-irritating compounds that can be hydrolyzed in vivo by pancreatic lipase. SFAEs can be synthesized by an esterification reaction between a sugar and a nonpolar fatty acid, both considered renewable sources. In this sense, lipase-catalyzed synthesis of SFAEs in mild reaction conditions must be considered a less harmful approach for the environment compared to current chemical synthesis, which is performed by adding an acyl chloride to a mixture of sugar, dimethylformamide (DMF), and pyridine [10]. The synthesis of SFAEs may be hampered by poor sugar solubility in most organic solvents, and this solubility becomes extremely low when the saccharide chain length increases. In addition, those polar solvents that can dissolve sugars are detrimental to enzyme activity and stability due to protein unfolding or the removal of essential water molecules required for lipase activity [11]. Only a few commercial lipases, such as immobilized lipase B from *Candida antarctica* (Novozyme 435), immobilized lipase from *Mucor miehei* (Lipozyme IM) [7], and lipase from *Pseudomonas stutzeri* [12], can be used to obtain high conversion yields in the synthesis of SFAEs, but the overall production cost is high. Thus, novel stable and regioselective lipases are required for the synthesis of different SFAEs and other GLs. Streptomycetes are industrially relevant Gram-positive filamentous bacteria with high G + C (>70%) genome content, which produce a wide variety of secondary metabolites with potent biological activities (e.g., antibiotics, immunosuppressors, pesticides, etc.) [13], as well as useful enzymes in biotechnology [14], including lipases for the synthesis of triglycerides enriched with long-chain n-3 polyunsaturated fatty acids [15,16], synthesis of SFAEs from glucose [17], as well as for the biodiesel [18] and detergent [19] industries.

Advances in genome sequencing and bioinformatics, as well as the large number of genome sequences deposited in public databases, have enabled the discovery of novel microbial enzymes to add to the portfolio of biocatalysts with special relevance in biotechnology, such as esterases [20], lipases [21], halohydrin dehalogenases [22], epoxidase hydrolases [23], cytochrome P450 monooxygenases [24], and hydroxynitrile lyases [25], among others. In the present work, we have performed an in silico search in the draft genome of *Streptomyces exfoliatus* DSMZ 41693 [26] to find lipases of biotechnological interest. Moreover, we report the heterologous expression and characterization of a novel lipase from this actinomycete (abbreviated as *Se*LipC). Interestingly, *Se*LipC is able to hydrolyze olive oil and several polyester-based plastics such as poly(butylene succinate), poly(butylene succinate)-*co*-(butylene adipate), and poly(ε-caprolactone). Likewise, promising results have been achieved in the synthesis of different SFAEs catalyzed by *Se*LipC. Our results demonstrate the versatile biotechnological applications of *Se*LipC, including the production of glycosylated lipids and the degradation of polyester-based plastics.

## 2. Results

### 2.1. Search, Cloning, and Expression of Novel Lipases from Streptomyces exfoliatus DSMZ 41693

**Figure 1:**
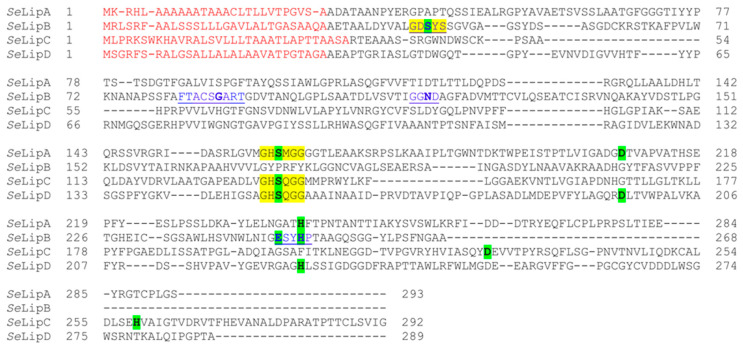
Sequence alignment of identified putative lipases in the draft genome of *Streptomyces exfoliatus* DSMZ 41693. The four lipases *Se*LipA, *Se*LipB, *Se*LipC, and *Se*LipD show a secretion signal peptide (in red) characteristic of *Streptomyces.* The lipase box of *Se*LipA, *Se*LipC, and *Se*LipD is indicated in yellow, as is the GDS(L)-like consensus motif I of *Se*LipB. The catalytic triad of all lipases is highlighted in bold green. Motifs I, II, III, and V of *Se*LipB are underlined in blue.

Four hypothetical *lip* genes (*lipA*, *lipB*, *lipC*, and *lipD*) encoding for novel lipases were identified in the draft genome of *S. exfoliatus* DSMZ 41693 [26] (GenBank: AZSS00000000): nt 152-1033 in contig 44 (*lipA*), nt 18904-19710 in contig 85 *(lipB*), nt 12616-13485 in contig 334 (*lipC*), and 48043-48921 in contig 355 (*lipD*). N-terminal secretion signals were predicted by the SignalP 4.1 server for all the deduced amino acid sequences [27], revealing that the identified putative lipases were extracellular enzymes. In addition, sequence alignment performed with COBALT [28] showed that *Se*LipB does not exhibit high overall sequence similarity to the other lipases (Figure 1). The 3D structure models predicted by RoseTTAFold [29] of three of these hypothetical lipases, hereafter referred to as *Se*LipA (WP_241775948.1), *Se*LipC (WP_024761750.1), and *Se*LipD (WP_024761024.1), show the typical α/β hydrolase fold (Figure S1A,C,D, Supplementary Material). Furthermore, their lipase box (G-H-S-X-G) and their catalytic triad were pinpointed (Figure 1). In contrast, the hypothetical tertiary fold of *Se*LipB (WP_024758087.1) differs from the α/β hydrolase fold (Figure S1B, Supplementary Material). In fact, *Se*LipB should be considered a member of the SGNH/GDSL hydrolase family [30] since it contains the GDS(L)-like consensus motif near the N-terminus (Figure 1) instead of the canonical lipase box. Additionally, active-site Ser of *Se*LipB is located in GDS motif I, whereas the highly conserved residues Gly, Asn (involved in the oxyanion hole), and the catalytic His are located in conserved motifs II, III, and V, respectively.

The four putative genes (including the signal peptide encoding sequence) were cloned into the expression vector pENV19 and recombinant strains of *Rhodococcus* sp. T104 harboring pENV19*Se*LipA, pENV19*Se*LipB, pENV19*Se*LipC, and pENV19*Se*LipD were obtained, as described in the Material and Methods section. Then, recombinant strains were grown at 30 °C for 72 h, and enzymatic activity was only detected in culture supernatants of the recombinant strains *Rhodococcus* pENV19*Se*LipA and pENV19*Se*LipC that produced *Se*LipA and *Se*LipC, respectively (Table S3, Supplementary Material), indicating that the signal peptide was correctly recognized by *Rhodococcu*s sp. T104 and confirming that native enzymes were expressed extracellularly. Both enzymes were able to hydrolyze several pNP esters, showing different specific activities. In fact, *Se*LipC showed approximately 2-fold higher activity than *Se*LipA on pNP-esters of fatty acids with acyl-chain lengths between 6 and 18 carbon atoms (Table S3, Supplementary Material). Considering these results, purification and characterization were exclusively focused on *Se*LipC.

### 2.2. Purification and Structural Characterization of SeLipC

**Figure 2:**
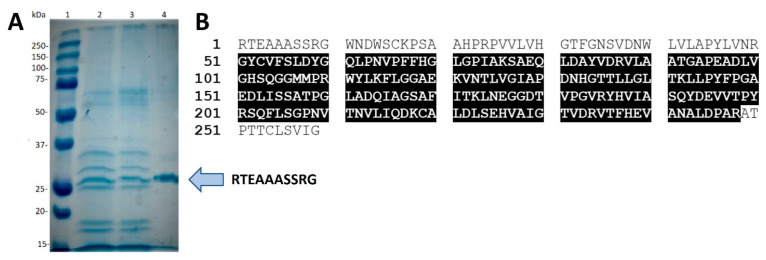
(**A**). SDS-PAGE analysis of purified *Se*LipC produced by the recombinant strain *Rhodococcus* pENV19*Se*LipC. Lane 1, standard molecular mass markers; lane 2, fermentation broth; lane 3, fermentation broth adjusted to 0.5 M ammonium sulfate; lane 4, purified *Se*LipC by HIC employing phenyl sepharose. The N-terminal sequence (indicated with an arrow) was determined by Edman’s degradation method. (**B**). Protein identification of *Se*LipC by peptide mass fingerprinting performed with MALDI-TOF mass spectrometry. A total of 76% of the mature *S*eLipC sequence was covered by peptide matches (shaded in black), as observed from MALDI-TOF analysis and MASCOT results (Figure S2, Supplementary Material).

Recombinant *Se*LipC was purified 7.2-fold (Table S4, Supplementary Material) from the cell-free broth by one-step hydrophobic interaction chromatography (HIC) using phenyl sepharose, as described in the Material and Methods section. The sequence of the pure protein band on SDS-PAGE gel (Figure 2A) was confirmed as lipase C from *Streptomyces exfoliatus* by peptide mass fingerprinting (Figure 2B).

**Figure 3:**
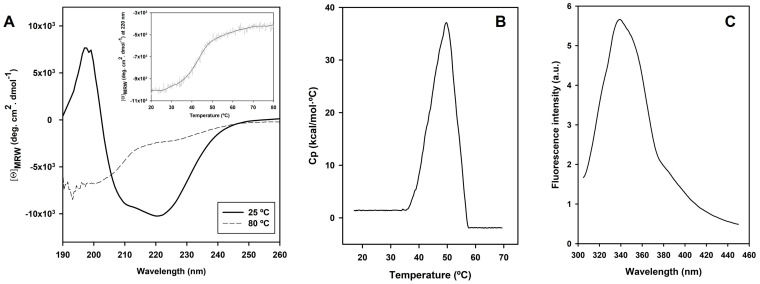
Structural characterization of pure *Se*LipC dissolved in 5 mM Tris/HCl buffer, pH 8.0. (**A**) Far-UV CD spectra were recorded with 0.29 mg/mL of lipase at 25 °C (solid line) and 80 °C (dashed line); inset: protein thermal unfolding studied by CD variation at 220 nm from 20 to 80 °C at a scan speed of 20 °C/h. (**B**) DSC thermogram was recorded with 0.8 mg/mL of lipase. (**C**) Fluorescence emission spectrum was recorded at 25 °C with 0.035 mg/mL of lipase.

Likewise, the N-terminal sequence (RTEAAASSRG) of the isolated protein band was also determined (Figure 2A). MALDI-TOF analysis of purified *Se*LipC (Figure S3, Supplementary Material) revealed a main peak of 27.1 kDa, which corresponds well to the theoretical molecular mass value (27.6 kDa). All these results indicate that the mature form of the enzyme is a monomer composed of 259 amino acids, and *Rhodococcus* correctly recognizes the secretion signal peptide of native *Se*LipC, which was composed of 33 amino acids as the one predicted by the SignalP server (Figure 1). After its purification, structural characterization of recombinant *Se*LipC was performed by spectroscopic and calorimetric studies (Figure 3).

On the one hand, the far-UV circular dichroism (CD) spectrum of *Se*LipC was recorded at 25 °C (Figure 3A), showing a negative ellipticity band at 220 nm. The secondary structure elements of the enzyme were determined by deconvolution of the CD spectrum, and results confirmed that native *Se*LipC contained 41% α-helix, 21% β-sheet, 16% β-turn, and 22% random coil, as expected for a folded α/β protein [31]. After gradual heating, thermal denaturation of the enzyme was confirmed, as deconvolution of the CD spectrum of *Se*LipC at 80 °C indicated an increment in the percentage of unordered structures and the disappearance of α-helix content. Differential scanning calorimetry (DSC) of *Se*LipC allowed us to observe an irreversible unfolding process with a single endothermic peak (Figure 3B). CD thermal denaturation of *Se*LipC revealed a melting temperature (*Tm*) of approximately 43.4 °C (inset Figure 3A), whereas a higher value (50.3 °C) was obtained after the analysis of the DSC thermogram (Figure 3B). Likewise, the specific enthalpy of the unfolding process could be calculated from the area of the DSC curve (*ΔH_cal_* = 1155 ± 389 kJ/mol). After excitation at 295 nm, a single peak at 340 nm was observed in the fluorescence emission spectrum of *Se*LipC (Figure 3C). Since there is no absorption by tyrosine at this wavelength, this intrinsic fluorescence should be attributed to the tryptophan residues of mature *Se*LipC. The value of the fluorescence maximum (340 nm) of *Se*LipC is like the one observed for other microbial lipases, despite their differences in the primary structure [32,33].

### 2.3. Biochemical Characterization of SeLipC

**Figure 4:**
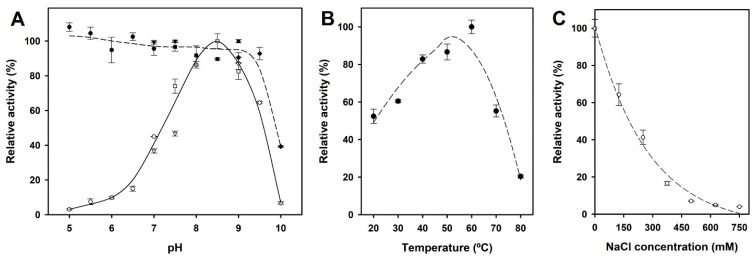
Effects of pH, temperature, and ionic strength on the activity and stability of *Se*LipC. (**A**) Effect of pH on enzyme activity (solid line) and stability (dotted line). The buffer systems at 50 mM were MES (pH 5.0–7.0) (circles), HEPES (pH 7.0–7.5) (triangles), Tris/HCl (pH 7.5–9.0) (squares), and glycine/NaOH (pH 9.0–10.0) (diamonds). (**B**) Effect of temperature on enzyme activity. (**C**) Effect of ionic strength on *Se*LipC activity. Assays were carried out in 50 mM Tris/HCl at pH 8.0 and 37 °C at different NaCl concentrations.

The effect of pH, temperature, and ionic strength on the activity and stability of *Se*LipC was studied using *p*-nitrophenyl palmitate (pNPP) as a substrate (Figure 4). On the one hand, hydrolysis of pNPP catalyzed by *Se*LipC was carried out in different buffer systems whose pH values ranged from 5 to 10. The lipase was found to be most active at pH 8.5; its activity was greatly reduced when pH was lower than 6.5, and it exhibited very low (<5%) of the optimal activity at pH 10.0 (Figure 4A). Likewise, stability was tested after incubation of lipase at different pH values for 10 min. Results show that the activity of *Se*LipC was abruptly decreased after incubation at pH 10.0, whereas it was stable when incubated at pH values ranging from 5.0 to 9.5 (Figure 4A). On the other hand, the purified enzyme displayed the highest activity at 60 °C (Figure 4B) but declined abruptly at temperatures above 70 °C. Likewise, *Se*LipC was sensitive to high ionic strength (*I*) as the enzyme lost 60% of its activity at 250 mM NaCl, while it was almost deactivated at salt concentrations higher than 500 mM (Figure 4C).

**Figure 5:**
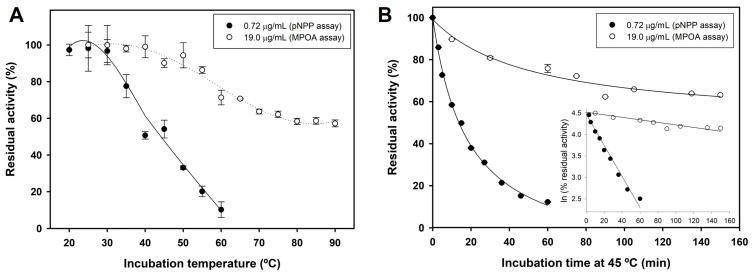
Thermal stability of *Se*LipC. (**A**) Effect of temperature on *Se*LipC stability using different enzyme concentrations and activity assays. (**B**) Thermal inactivation kinetics of *Se*LipC at 45 °C using different enzyme concentrations and activity assays; inset: the decrease in enzyme activity followed exponential regression (first-order kinetics). Conditions are detailed in the Materials and Methods section.

Finally, the thermal stability of *Se*LipC was investigated by incubating lipase at different temperatures for 10 min in 15 mM Tris/HCl pH 8.0. As depicted in Figure 5A, *Se*LipC shows very poor thermal stability at low protein concentrations (0.72 µg/mL) since residual enzyme activity was strongly decreased from 35 to 60 °C. However, its thermostability was clearly enhanced when protein concentration was increased approximately 25-fold (19 µg/mL), allowing the enzyme to maintain 60% residual activity after incubation at temperatures above 70 °C.

To confirm this observation, the thermal inactivation kinetics of *Se*LipC at 45 °C were studied at different enzyme concentrations. The depicted deactivation curves (Figure 5B) followed single exponential decay, and therefore, *Se*LipC showed first-order deactivation kinetics with both enzyme concentrations at 45 °C. As postulated, the enzyme at low protein concentration exhibited a quicker deactivation pattern (*k_d_* = 0.0362 min^−1^ and *t*_1/2_ = 19 min) than the one observed at higher protein concentration (*k_d_* = 0.00296 min^−1^ and *t*_1/2_ = 234 min).

**Figure 6:**
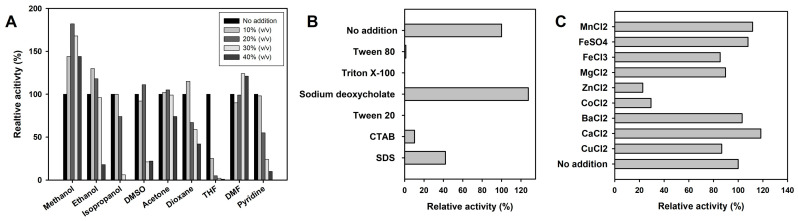
Effects of different additives on SeLipC activity. (**A**) water-miscible cosolvents at different concentrations. (**B**) Detergents at 0.2% (*w*/*v*) concentration. (**C**) Metal ions at 1 mM concentration. The data were the means of three independent experiments. More details are in the Materials and Methods section.

In addition, the effect of different additives (reducing agents, organic cosolvents, detergents, and metal ions) on *Se*LipC activity was studied using the pNPP assay. On the one hand, *Se*LipC was not inhibited by the reducing agent’s dithiothreitol (DTT) and 2-mercaptoethanol (Table S5, Supplementary Material). On the other hand, the presence of different amounts of water-miscible organic cosolvents affected the activity of *Se*LipC. The organic solvents selected were several aprotic polar solvents such as dimethyl sulfoxide (DMSO), dioxane, dimethylformamide (DMF), acetone, tetrahydrofuran (THF), and pyridine, as well as alcohols (methanol, ethanol, and isopropanol) whose final concentration varied from 10 to 40% (*v*/*v*) in the assay volume (Figure 6A). Aprotic polar cosolvents at 10% (*v*/*v*) concentration did not affect *Se*LipC activity, except THF, which drastically reduced enzyme activity and nearly abolished it at higher concentrations. Acetone and DMF did not affect enzyme activity noticeably at the employed concentrations, whereas DMSO, dioxane, and pyridine caused a concentration-dependent loss of *Se*LipC activity. Regarding alcohols, enzyme activity was enhanced by methanol at all concentrations and by ethanol up to a 20% (*v*/*v*) concentration, but it was gradually reduced at increasing concentrations of isopropanol.

Moreover, the enzymatic activity displayed by *Se*LipC in the presence of different detergents at a 0.2% (*w*/*v*) concentration was compared, as indicated in Figure 6B. Sodium deoxycholate enhanced enzyme activity, whereas non-ionic detergents such as Triton X-100, Tween 20, and Tween 80 abolished it. Other ionic surfactants, such as SDS and CTAB, produced a strong inhibitory effect. The effect of various metal ions at 1 mM concentration on *Se*LipC activity was also assessed (Figure 6C). Enzyme activity was moderately reduced by the presence of Cu^2+^, Mg^2+^, and Fe^3+^ (85–90%), but strongly diminished by Zn^2+^ and Co^2+^ (23% and 29%, respectively). Other metal ions (such as Ba^2+^, Ca^2+^, Mn^2+^, and Fe^2+^) showed a slight activation effect on lipase activity.

**Figure 7:**
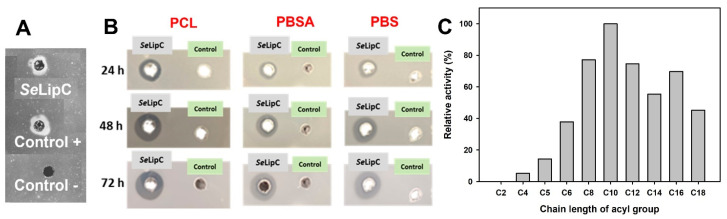
Substrate specificity of *Se*LipC. (**A**) Rhodamine plate assay for lipase activity detection using olive oil as substrate. *Se*LipC sample (40 μL at 0.043 mg/mL) was loaded in 5-mm diameter wells made in the agar plate and incubated at 37 °C for 24 h. Lipase TL from Meito Sangyo was loaded as a positive control (40 μL at 0.5 mg/mL), and 15 mM Tris/HCl buffer pH 8 was loaded as a negative control. (**B**) Agar plate assay for the detection of hydrolytic activity against different polyester-type plastics: (1) PCL: poly(ε-caprolactone), (2) PBSA: poly(butylene succinate)-*co*-(butylene adipate), and (3) PBS: poly(butylene succinate). *Se*LipC sample (40 μL at 0.043 mg/mL) was loaded in 5-mm diameter wells made in the agar plate and incubated at 37 °C for 24, 48, and 72 h. A total of 40 μL buffer (15 mM Tris/HCl pH 8) was loaded as a negative control. (**C**) Relative activity of *Se*LipC towards several *p*-nitrophenyl alkanoate esters. More details are in the Materials and Methods section.

To assess if SeLipC was able to degrade triglycerides, an agar plate assay was carried out using olive oil as a substrate. This method allows for the detection of lipase activity on triacylglycerols present in olive oil since released fatty acids interact with rhodamine dye, producing an orange fluorescence that can be detected by UV irradiation [34]. In our case, a fluorescence halo was observed on the agar plate (Figure 7A), indicating that SeLipC was a true lipase. On the other hand, SeLipC was also able to degrade different polyester-type plastics such as PBS, PBSA, and PCL, as shown by the appearance of clearance halos on agar plates containing the corresponding emulsified polymer (Figure 7B). The diameter of the halos was monitored after 24, 48, and 72 h of incubation at 37 °C. In the case of PBS and PBSA plates, the halo diameters were gradually increased up to 72 h, whereas the observed halo diameter in the PCL plate was no further increased at higher times than 48 h.

Finally, the substrate specificity of SeLipC was studied using several p-nitrophenyl alkanoate esters of varying alkyl chain lengths. As shown in Figure 7C, SeLipC showed the highest activity using pNP-decanoate (C10), whereas its relative activity was significantly reduced employing esters with shorter-length acyl chains (from C2 to C6). Likewise, the preferred substrates of SeLipC were long-chain p-nitrophenyl esters whose acyl chain length was greater than 12 carbon atoms. In the case of pNPP (C16), a steady-state kinetic study was performed to determine the kinetic parameters. According to the hyperbolic regression analysis (Figure S4, Supplementary Material), SeLipC showed the following kinetic parameters: *K_M_* = 49.6 ± 11.2 µM, *k_cat_* = 57 s^−1^ (*V_max_* = 124 µmol·min^−1^·mg^−1^), and *k_cat_*/*K_M_* = 1.15 × 10^6^ s^−1^·M^−1^.

### 2.4. Synthesis of Glycosylated Lipids Catalyzed by SeLipC

**Figure 8:**
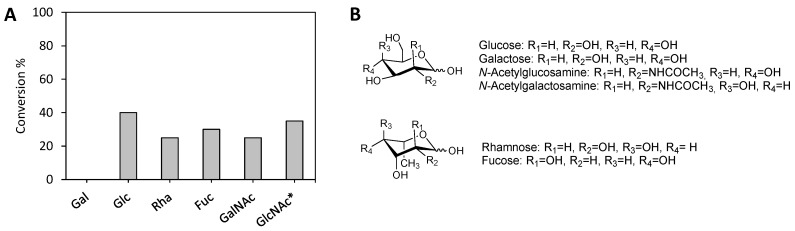
(**A**) Conversions were achieved in the transesterification reaction catalyzed by *Se*LipC of different sugars and vinyl laurate after 48 h. * 24 h reaction time. (**B**) Sugars are employed as substrates. Conditions are detailed in the Materials and Methods section.

The potential use of *Se*LipC in the synthesis of SFAEs was also investigated. Therefore, commercial sugars (Glc, Gal, Rha, Fuc, GlcNAc, and GalNAc) were tested as substrates in the transesterification reaction mediated by *Se*LipC, using vinyl laurate as an acyl donor and anhydrous THF as solvent. As shown in Figure 8, no product could be detected in the transesterification reaction of galactose (Gal). 

In contrast, 40% conversion was obtained with glucose (Glc) as substrate, although a mixture of different monoesterified products has been observed since the first 6 h. In addition, when rhamnose (Rha) and fucose (Fuc) were employed as sugar substrates, 25% and 30% conversions were, respectively, achieved to produce a single monoester laurate. Finally, the transesterification of *N*-acetylgalactosamine (GalNAc) afforded 25% conversion after 48 h, and 35% conversion was achieved in the case of *N*-acetylglucosamine (GlcNAc) after just 24 h, although it did not considerably progress after that time. 

## 3. Discussion

*Streptomyces exfoliatus* DSMZ 41693 is a Gram-positive actinomycete whose genome [26] contains genes that encode for many putative hydrolytic enzymes such as cellulases, amylases, xylanases, chitinases, proteases, lipases, and esterases. In this sense, four putative genes encoding lipases (*lipA*, *lipB*, *lipC*, and *lipD*) were cloned and expressed using shuttle vector pENV19 and *Rhodococcus* sp. T104. The suitability of this system relies mainly on the similar codon usage of several actinomycetes and its ability to recognize and cleave native signal peptides, as reported for the expression of some polyhydroxyalkanoate depolymerizes from *Streptomyces exfoliatus* [35,36] and *Streptomyces ascomycinicus* [37], as well as an *N*-acylhomoserine lactone acylase from *Actinoplanes utahensis* [38]. In this work, successful extracellular production of two lipases (*Se*LipA and *Se*LipC) from *S. exfoliatus* has been achieved since enzymatic activity was detected in culture broths of recombinant *Rhodococcus* sp. T104 harbouring pENV19*Se*LipA and pENV19*Se*LipC plasmids (Table S3, Supplementary Material). Once *Se*LipC was selected according to its preliminary promising results, enzyme purification was successfully performed by one-step hydrophobic interaction chromatography (HIC) followed by isopropanol gradient elution (Table S4, Supplementary Material), a widely reported method for lipase purification [39]. Results of the N-terminal amino acid sequence and peptide mass fingerprinting of pure *Se*LipC (Figure 2) confirmed that *Rhodococcus* sp. T104 produced extracellularly the mature and active enzyme after cleaving a secretion signal peptide of 33 amino acids (Figure 1). Again, our expression system in *Rhodococcus* sp. T104 has demonstrated its versatility to produce extracellularly different recombinant enzymes from actinomycetes.

**Figure 9:**
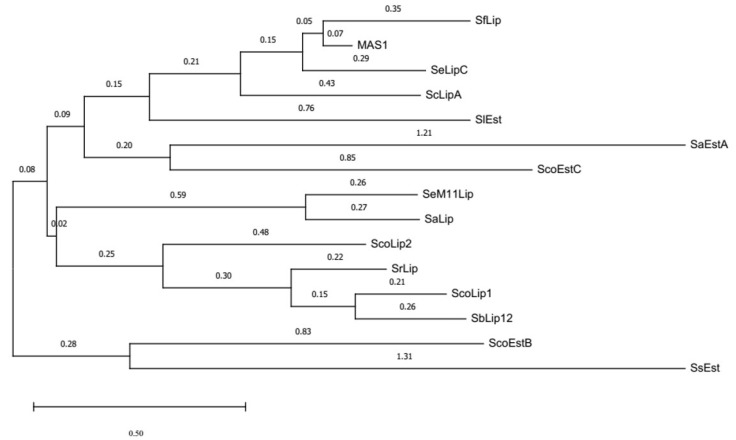
Phylogenetic tree of *Se*LipC, including other lipases and esterases from *Streptomyces* that have already been characterized (NCBI accession code). ScoLip1: lipase 1 from *S. coelicolor* A3(2) (Q9S2A5.1) [53,54]; ScoLip2: lipase 2 from *S. coelicolor* A3(2) (Q93J06.1) [53]; SeM11Lip: Lipase from *S. exfoliatus* M11 (AAB51445.1) [55,56]; SrLip: lipase from *S. rimosus* (AF394224_1) [52,57]; SaLip: lipase from *S. albus* (AAA53485.1) [58]; SfLip: lipase from *S. fradiae* (ABR14271.1) [42]; ScLipA: lipase from *S. cinnamomeus (*AAB71210.1) [43]; MAS1: lipase from *Streptomyces* sp. W007 (EHM30041.1) [44]; SbLip12: GSLD lipase from *S. bacillaris* (DAD54739.1) [59]; SaEstA: esterase A from *S. anulatus* (formerly *S. chrysomallus*) (CAA78842.1) [60]; ScoEstB: esterase B from *S. coelicolor* A3(2) (CAB89027.1) [61]; ScoEstC: esterase C from *S. coelicolor* A3(2) (NP_629313.1) [62]; SlEst: esterase from *S. lividans* (ACX47356.1) [63]; SsEst: esterase from *S. scabies* [64]. The evolutionary history was inferred using the neighbor-joining method. The tree is drawn to scale, with branch lengths (next to the branches) in the same units as those of the evolutionary distances used to infer the phylogenetic tree. The evolutionary distances were computed using the Poisson correction method and are in units of the number of amino acid substitutions per site. This analysis involved 15 amino acid sequences. All ambiguous positions were removed for each sequence pair (pairwise deletion option). There were a total of 95 positions in the final dataset. Evolutionary analyses were conducted in MEGA11 [65].

Based on a phylogenetic tree constructed with reported sequences of characterized lipases and esterases from different *Streptomyces* species (Figure 9), *Se*LipC could be assigned to group 7 within the family I of lipolytic enzymes. In this sense, lipolytic enzymes are currently classified into 19 families based on phylogenetic criteria, conserved motifs, and biological functions [40,41]. Moreover, family I consists of eight subfamilies that comprise all true lipases (meaning triacylglycerol hydrolases), since these enzymes can hydrolyze triglycerides into fatty acids and glycerol, among other features. It is worth mentioning that *Se*LipC shares a close common ancestor with other lipases from different species of *Streptomyces*, such as lipase from *S. fradiae* var. k11 (reported as lipS221) [42], lipase from *S. cinnamomeus* Tü89 [43], and thermostable lipase from marine *Streptomyces* sp. W007 (reported as MAS1) [44], all of which are classified as members of subfamily I.7 [41]. Moreover, *Se*LipC shares the highest sequence identity (81.9%) with MAS1 lipase, whose crystal structure has been recently determined [45].

The optimum pH for *Se*LipC to hydrolyze pNPP is 8.5 (Figure 4A), a value close to the one described (pH 8.0) for lipases from other actinomycetes such as *Amycolatopsis mediterranei* DSM 43304 [46], *Streptomyces violascens* OC125-8 [47], *Streptomyces bambergiensis* OC 25-4 [48], *Streptomyces* sp. OC 119-7 [49], *Streptomyces* sp. CS268 [50], and *Streptomyces* sp. CS273 [51], but far to those for alkaline lipases from *Streptomyces cellulosae* AU-10 (pH 9.0) [19] and *S. fradiae* var. k11 (pH 9.8) [42] (Table 1). The high stability of *Se*LipC at a broad pH range (5~9.5) is comparable to the one reported for lipases from *S. rimosus* (4~10) [52], *S. fradiae* var. k11 (4.0~10.0) [42], and *S. coelicolor* A(3)2 (6.0~9.0) [53], making our lipase also applicable at alkaline pH conditions.

On the other hand, *Se*LipC showed the highest activity at 60 °C using pNPP as substrate (Figure 4B), like other lipases from actinomycetes such as *A. mediterranei* [60], *S. fradiae* var. k11 [42], *Streptomyces coelicolor* A3(2) [54], and *Streptomyces rimosus* [52], which display the highest activity at approximately 55–60 °C (Table 1). Thermal deactivation kinetics of *Se*LipC were studied using two different activity assays depending on the enzyme concentration employed during incubation at 45 °C: the chromogenic pNPP assay for a low enzyme concentration (0.72 µg/mL), whereas MPOA esterase assay for high enzyme concentration (19 µg/mL) (Figure 5). Noteworthy is that the half-life of *Se*LipC at 45 °C was significantly improved when protein concentration was increased (Figure 5A,B). This result is in accordance with the different melting temperatures (*T_m_*) estimated by circular dichroism (43.4 °C) and calorimetry (50.3 °C), whose distant values could be attributed to the lower enzyme concentration employed during the CD experiment compared to the DSC experiment (Figure 3A,B, respectively). In addition, the aggregation state of *Se*LipC was also confirmed by analytical ultracentrifugation studies (Figure S5, Supplementary Material). Lipases have a general trend to self-assemble in solution since they have hydrophobic areas surrounding their active center that come into close contact with the concomitant hydrophobic areas of other lipase molecules, as shown by X-ray diffraction-deduced 3D structures in the form of dimers [66]. In fact, the mechanism underlying lipase-lipase dimerization is thought to be based on interactions between the hydrophobic lid domain and hydrophobic cleft of lipases [67]. In addition, several lipases (those from *Alcaligenes* sp., *Candida rugosa*, *Mucor miehei*, *Pseudomonas fluorescens*, and *Thermomyces lanuginosus*) have been described to show different apparent molecular weights in gel filtration experiments at different protein concentrations [68,69]. Such an aggregation state of these lipases has effects on lipase activity and stability, so the higher the protein concentration, the higher the stability [67]. Furthermore, aggregation would be a characteristic thermostability strategy of lipases from thermoalkalophilic microorganisms such as *Bacillus thermocatenulatus*. In this enzyme, a conserved tryptophan (W211) in the lid contributes not only to intramolecular interactions at the subunit interface but also to the improvement of thermostability via aggregation [70]. In lipase from *Thermomyces lanuginosus*, a tryptophan residue located in the lid (W89) is responsible for 60% of the fluorescence emission because of the low fluorescence emission of the other three tryptophan residues (W117, W221, and W260) [71]. In the case of *Se*LipC, there are four tryptophan residues (W11, W14, W40, and W111), which should be mainly responsible for the fluorescence emission of the protein (Figure 3C), but none of them would be positioned in the lid covering the active site. This affirmation is supported by the fact that the lid domain of MAS1 lipase (G140–T164) [45] shares a high identity (75%) with the sequence G134–T158 of mature *Se*LipC (Figure 1), which might be considered as its lid (Figure S1C in Supplemental Material). As discussed earlier, this lid sequence of 24 amino acid residues contains no tryptophan residues, and other aromatic residues inside (such as Y146 and F147) would not contribute to the emission fluorescence (Figure 3C). On the other hand, *Se*LipC was subjected to chemical modification with phenylmethanesulfonyl fluoride (PMSF), an active site serine-specific modification reagent. Enzymatic activity with pNPP was significantly decreased when PMSF concentration was increased from 1 to 10 mM (84.7% and 51.2% residual activity, respectively) (Table S6, Supplementary Material). In contrast, *Se*LipC was resistant to inactivation by PMSF at both concentrations in the presence of the substrate (100% and 90.2% residual activity, respectively), indicating that the active site was protected against chemical modification. These results suggest that a serine residue located at the active site of the enzyme is involved in the activity of *Se*LipC; it could be the putative catalytic serine identified in the lipase box (G-H-S-X-G) (Figure 1). However, directed mutagenesis of this residue should be addressed to confirm this hypothesis. PMSF has been reported to produce similar inhibitory effects on lipases from *Streptomyces bambergiensis* [48], *Streptomyces* sp. CS133 [50], and *Streptomyces* sp. CS273 [51], among others.

The alteration of activity and stability of lipases in non-aqueous media has important implications for their potential applications in biotechnology [72]. Therefore, the effect of various water-miscible organic cosolvents on *Se*LipC activity was investigated (Figure 6A), keeping in mind its possible application in biotransformations of non-natural substrates, which often have a low solubility in water. Previously, the presence of 10% (*v*/*v*) isopropanol was established as the condition for 100% relative activity since this cosolvent was needed for the standard assay conditions to solubilize the pNPP substrate. Enzyme activity was enhanced in the presence of increasing concentrations of methanol up to 40% (*v*/*v*) and ethanol up to 20% (*v*/*v*). Likewise, the presence of some aprotic solvents also yielded enhanced activity, such as DMF at 20% (*v*/*v*) and DMSO at 30–40% (*v*/*v*). Taking into consideration the high hydrophobicity of pNPP, this enhanced activity might be related to an improved solubilization of this substrate in the reaction mixture. Moreover, enzyme activation in the presence of water-miscible solvents is a rare property that has been reported in a few actinomycete lipases [46,54,73]. On the contrary, there are some other cosolvents that inhibit *Se*LipC activity, such as THF, dioxane, and isopropanol, at concentrations greater than 20% (*v*/*v*). In this case, such behavior should be related to the extreme denaturing capacity (DC) of these cosolvents according to the scale of Khmelnitsky [74] (DC values of 100, 92.1, and 70.2, respectively), a parameter that has been proven correct to predict enzyme stability in water-organic cosolvent monophasic systems [75]. In addition, an inhibitory effect on *Se*LipC activity was observed in the presence of different surfactants (Figure 6B). *Se*LipC activity was abruptly decreased in the presence of cationic surfactant CTAB as well as non-ionic surfactants such as Tween 20, Tween 80, and Triton X-100. In the case of anionic surfactants, 127% relative activity was obtained when sodium deoxycholate was employed in the assay, whereas the activity was reduced three-fold in the presence of SDS. A similar behavior was observed in lipase from *Streptomyces* sp. CS273, although its activity was enhanced in the presence of Triton X-100 [51], a detergent that was detrimental to *Se*LipC. In this sense, surfactants play a key role in lipase activity since these molecules can preferentially interact with their lids through hydrophobic bonds, inducing conformational changes in protein structure [76]. As a rule of thumb, non-ionic and zwitterionic surfactants have hydrophobic interactions with lipases, whereas the interactions of anionic and cationic surfactants with lipases are mainly electrostatic [77]. Likewise, surfactants may cause micelle formation at a given concentration, promoting a suitable microenvironment for the hydrolysis of hydrophobic substrates in aqueous solutions such as triglycerides [78]. In the case of *Se*LipC, sodium deoxycholate would stabilize the pNPP emulsion and create the appropriate micellar environment for enzyme catalysis as described for pancreatic lipase [79]. Regarding the effect of some metal ions, *Se*LipC activity was not significantly enhanced by the addition of Ca^2+^ (Figure 6C). Since the Ca^2+^-binding motif GXXGXD [80] was not found in the enzyme sequence (Figure 1), *Se*LipC should not be considered a Ca^2+^-dependent lipase. In addition, the inhibitory activity of Zn^2+^ and Co^2+^ might be due to direct inhibition of the catalytic site and/or formation of complexes between these transition metal ions and the ionized palmitate (a product of pNPP hydrolysis), as described for other microbial lipases [81,82]. In addition, we examined the effect of reducing agents on lipase activity (Table S5, Supplementary Material). As observed, *Se*LipC was not inhibited by DTT and 2-mercaptoethanol. Our results suggest that those cysteine residues suspected to be involved in disulfide bonds in *Se*LipC (C16-C53 and C219-C254, Figure 1) are not needed for activity but probably for protein folding and stability.

The substrate specificity of *Se*LipC was studied with *p*-nitrophenyl alkanoate esters (Figure 7C). The highest hydrolysis rate was obtained with pNP-decanoate, followed by long-chain *p*-nitrophenyl esters whose acyl chain lengths were higher than 10 carbon atoms. Likewise, *Se*LipC showed activity using olive oil as a substrate (Figure 7A), which could be due to its high content of long unsaturated fatty acyl chains such as oleic acid. All these results would confirm that *Se*LipC is a true lipase belonging to subfamily I.7 [41]. This subfamily would include other lipases from actinomycetes that share a high sequence identity (not including their signal peptide) with mature *Se*LipC, such as those from *S. cinnamomeus* Tü89 (44.9%), *S. fradiae* var. k11 (77.2%), and *Streptomyces* sp. W007 (81.9%). In addition, the kinetic parameters of *Se*LipC were determined using pNPP as a substrate (Figure S4, Supplementary Material). The determined values were different than the ones described for other microbial lipases using the same substrate (Table 1). Such differences should be attributed to the dissimilar amino acid sequences of these lipases as well as to the different assay conditions that were employed for the determination of enzyme activity in each case.

**Table 1:** Comparison of biochemical features of lipase C from *Streptomyces exfoliatus* DSMZ 41693 (*Se*LipC) with other reported microbial lipases.

Lipase Source	Maximum Activity at		Kinetic Parameters Using pNPP as a Substrate				Reference
	T (°C)	pH	*V_max_* (IU/mg)	*K_M_* (µM)	*k*_cat_ (s^−1^)	*k*_cat_/*K_M_* (s^−1^ M^−1^)	
**Actinomycetes**							
*Amycolatopsis mediterranei* DSM 43304	60	8.0	2.53 × 10^3^	99	n.d.	n.d.	[46]
*Streptomyces bambergiensis* OC 25-4	50	8.0	n.d.	n.d.	n.d.	n.d.	[48]
*Streptomyces cellulosae* AU-10	40	9.0	n.d.	340	n.d.	n.d.	[19]
*Streptomyces coelicolor* A3(2)	55	8.0	n.d.	n.d.	n.d.	n.d.	[54]
*Streptomyces fradiae* var. k11	55	9.8	436.6	139	n.d.	n.d.	[42]
*Streptomyces rimosus* R6-554W	55	9.5	n.d.	n.d.	n.d.	n.d.	[52]
*Streptomyces* sp. CS133	40	7.5	n.d.	152	n.d.	n.d.	[81]
*Streptomyces* sp. CS268	30	8.0	206 × 10^3^	2920	n.d.	n.d.	[50]
*Streptomyces* sp. CS273	40	8.5	n.d.	n.d.	n.d.	n.d.	[51]
*Streptomyces* sp. CS326	40	7.5	4.6 × 10^3^	240	n.d.	n.d.	[83]
*Streptomyces* sp. OC 119-7	50	8.0	n.d.	n.d.	n.d.	n.d.	[49]
*Streptomyces* sp. W007	40	7.0	n.d.	n.d.	n.d.	n.d.	[44]
*Streptomyces violascens* OC125-8	40	8.0	600	259	n.d.	n.d.	[47]
*Streptomyces exfoliatus* DSMZ 41693	60	8.5	124	49.6	57	1.15 × 10^6^	This study
**Other microorganisms**							
*Bacillus thermoamylovorans* BHK67	55	7.5	n.d.	7720	227	2.94 × 10^4^	[84]
*Burkholderia multivorans* V2	45	8.0	5.62	1560	n.d.	n.d.	[85]
*Cellulomonas flavigena*	30	7.0	n.d.	1330	7.22	5.43 × 10^3^	[86]
*Pseudomonas punonensis*	30	9.0	n.d.	160	3.48	1.79 × 10^4^	[87]
*Rhizopus chinensis*	40	8.5	n.d.	304	18.9	6.22 × 10^4^	[88]
*Yarrowia lipolytica*	40	8.0	n.d.	108	158	1.53 × 10^6^	[89]

n.d.: not determined.

The bioremediation potential of microbial lipases has emerged as one solution to degrade polyester-based plastics together with other enzymes such as cutinases, polyhydroxyalkanoate depolymerizes, and other hydrolases [90]. In this sense, *Se*LipC is able to degrade several polyester-type polymers, such as PBS, PBSA, and PCL, within 3 days (Figure 7B). Similarly, some microbial lipases can hydrolyze films of the same polymers within a few days or hours [91,92,93,94,95], typically following a surface degradation mechanism.

Interestingly, *Se*LipC can catalyze the enzymatic synthesis of different sugar fatty acid esters by transesterification reactions using vinyl laurate as an acyl donor. In this sense, the selection of an appropriate solvent is one of the critical parameters in the preparation of SFAEs since it must solubilize two substrates of very different polarity (the sugar and the fatty acid ester) without the presence of water. As THF had previously been shown to be an adequate solvent in the enzymatic synthesis of esters of Rha catalyzed by lipase from *P. stutzeri* [12], it was selected to carry out this preliminary study with *Se*LipC. Despite THF at 10–30% (*v*/*v*) having a detrimental effect on the hydrolytic activity of *Se*LipC towards pNPP (Figure 6A), we presumed that the same enzyme might catalyze the synthesis of SFAEs in anhydrous THF since the absence of water in the solvent would avoid reversion of the synthetic reaction [12], as well as protein denaturation. This last assumption was supported by the fact that some lipases that denature in a system composed of a homogeneous mixture of an aprotic solvent and water are stable in the same totally anhydrous solvent system. This behavior is due to the increasing rigidity of the lipase structure, which increases its stability, although it may hinder its activity [11]. Our hypothesis was confirmed, and lyophilized *Se*LipC performed successfully in anhydrous THF during the synthesis of laureate esters from different sugars with moderate yields: Glc (40%), Rha (25%), Fuc (30%), GlcNAc (35%), and GalNAc (25%) (Figure 8). In the case of Rha and its epimer Fuc, our results are very promising since rhamnolipids have gained great attention in recent years due to their surfactant properties as well as their antimicrobial and anticancer activities. Raku and Tokiwa performed the enzymatic esterification of these 6-deoxysugars employing divinyl adipate as an acyl donor [96]. In that work, lipase P from *Pseudomonas* sp. allowed the monoesterification of Rha in pyridine to reach 59% conversion, and *Bacillus subtilis* protease was the enzyme that selectively recognized Fuc to achieve 72% conversion in DMF. To the best of our knowledge, *Se*LipC is the first lipase described to obtain 6-deoxysugar esters of both Rha and Fuc. Furthermore, aminosugars such as GlcNAc and GalNAc have been scarcely employed as substrates for lipases in the preparation of GLs in a satisfactory manner, making *Se*LipC a new enzymatic tool for their synthesis. Pöhnlein et al. described the transesterification of GlcNAc mediated by immobilized lipase B from *Candida antarctica* (Novozyme 435), affording just 0.8% yield. It was necessary to increase the hydrophobicity of the sugar by employing N-butyrylglucosamine to achieve a 30% yield [97]. Our results should be improved after optimization of the reaction conditions during SFAE synthesis, as well as solvent engineering and trying to use environmentally friendly organic media. Moreover, *Se*LipC stabilization against thermal deactivation and solvent denaturation might be achieved through enzyme immobilization and protein engineering. In this sense, both approaches have been successfully worked out in the stabilization of MAS1 lipase isolated from *Streptomyces* sp. strain W007 [16,98,99,100].

## 4. Materials and Methods

### 4.1. Materials

Cell culture media were from Becton Dickinson (Franklin Lakes, NJ, USA). Thermo Fisher Scientific Inc. (Pittsburgh, PA, USA) provided potassium phosphate, Tris/HCl, sodium citrate, sodium chloride, hydrochloric acid, orthophosphoric acid, and organic solvents. Kanamycin, MES, HEPES, methyl phenoxyacetate (MPOA), phenoxyacetic acid (POA), glycine, acrylamide, *N*,*N*′-methylenebisacrylamide, bromophenol blue, Coomassie Brilliant Blue G-250, amido black, detergents, salts, olive oil, phenylmethanesulfonyl fluoride (PMSF), dithiothreitol (DTT), 2-mercaptoethanol, sodium *N*-lauroylsarcosinate (sarkosil NL), *p*-nitrophenyl (pNP) esters with varying acyl chain lengths from C2 to C18, glucose (Glc), galactose (Gal), rhamnose (Rha), fucose (Fuc), *N*-acetylglucosamine (GlcNAc), *N*-acetylgalactosamine (GalNAc), and vinyl laurate were purchased from Sigma-Aldrich (St. Louis, MO, USA). Poly(butylene succinate) (PBS) and poly(butylene succinate)-*co*-(butylene adipate) (PBSA) were purchased from Showa Denko K.K. (Tokyo, Japan) under the trade names Bionolle 1020MD and Bionolle 3020MD, respectively. Poly(ε-caprolactone) (PCL) was obtained from TRESNO (Poland). Restriction enzymes were purchased from New England Biolabs (Ipswich, Massachusetts, USA), and oligonucleotides were synthetized by Sigma-Aldrich (St. Louis, MO, USA).

### 4.2. Bacterial Strains, Culture Media, and Culture Conditions

Bacterial strains, plasmids, and oligonucleotides used are listed in Table S1 (Supplementary Material). *S. exfoliatus* DSMZ 41693 was used as a chromosomal DNA source [26,36]. *Escherichia coli* DH5α was used as the host for subcloning experiments [101], and *Rhodococcus* sp. T104 KACC 21099 was used as a host for gene expression [35,102]. Cells of recombinant *Rhodococcus* strains were grown on 2×YTG medium at 30 °C supplemented with 100 µg/mL kanamycin as described [35]. Plasmid purifications were performed using the High Pure Plasmid Isolation Kit from Roche (Mannheim, Germany). Plasmid DNA was sequenced according to Sanger [103] by BigDye Terminator v3.1 with an automatic DNA sequencer, ABI Prism 3730 (Applied Biosystems Inc., Waltham, MA, USA), in Secugen S.L. (Madrid, Spain).

### 4.3. Cloning and Heterologous Expression of Lip Genes from S. exfoliatus by Rhodococcus sp. T104

In silico search of *lip* genes in the draft genome of *S. exfoliatus* DSMZ 41693 [26] (GenBank: AZSS00000000) was carried out with the tBLASTn tool of the BioEdit program and the amino acid sequence of *Burkholderia cepacia* lipase (UniProt P22088) as reference. Hypothetical genes encoding lipases from *S. exfoliatus* DSMZ 41693, including their signal peptide coding sequence (*lipA*, *lipB*, *lipC*, and *lipD*), were amplified by PCR using Phusion DNA polymerase (New England Biolabs, EEUU) and *S. exfoliatus* DSMZ 41693 genomic DNA as template. *Xba*I and *EcoR*I restriction sites were included in the primers (Table S1, Supplementary Material) in order to clone the PCR product in the pENV19 shuttle vector [35]. Each PCR was performed in Mastercycler Personal equipment (Eppendorf, Germany) using a reaction mix containing 0.5 µM of each oligonucleotide pair, 0.25 mM dNTPs, 4% (*v*/*v*) DMSO, 10 ng/µL DNA template, and 0.02 U Phusion DNA polymerase in Phusion HF or GC buffer (New England Biolabs, USA). Reaction conditions are indicated in Table S2 (Supplementary Material). The resulting recombinant plasmids pENV19*Se*LipA, pENV19*Se*LipB, pENV19*Se*LipC, and pENV19*Se*LipD, harboring the lipase genes *lipA*, *lipB*, *lipC*, and *lipD*, respectively, were used to transform chemocompetent *E. coli* DH5α cells [104] and electrocompetent *Rhodococcus* sp. T104 cells [35]. Recombinant lipases were produced by culturing cells of recombinant *Rhodococcus* strains at 30 °C for 72 h and 250 rpm, as described above.

### 4.4. Purification of Recombinant SeLipC from Streptomyces exfoliatus

The purification of recombinant *Se*LipC produced by *Rhodococcus* T104 pENV19*Se*LipC was carried out through a single hydrophobic chromatographic step from an 800 mL culture broth. Cells were discarded by centrifugation at 8500*× g* for 20 min at 4 °C, and the supernatant was subsequently filtered by Whatman^®^ filter paper CHR 3 mm Cytiva. Then, (NH_4_)_2_SO_4_ was added to the cell-free culture broth at a final concentration of 0.5 M, centrifuged at 17,500*× g* for 20 min at 4 °C, and the supernatant was applied into a column packed with 25 mL of phenyl sepharose Fast Flow (GE Healthcare, Chicago, IL, USA) equilibrated with 15 mM Tris/HCl, 0.5 M (NH_4_)_2_SO_4_, pH 8.0 buffer. Hydrophobic interaction chromatography (HIC) was performed using an FPLC NGC Chromatography System (Bio-Rad, Hercules, CA, USA) at room temperature with a flow rate of 1.5 mL/min. After washing with 100 mL of equilibration buffer, the enzyme was eluted with a 50 mL linear gradient of isopropanol from 0 to 20% (*v*/*v*) in 15 mM Tris/HCl buffer at pH 8.0, followed by 200 mL of isopropanol at 20% (*v*/*v*) in the same buffer. Fractions with lipase activity were pooled, and protein concentration was determined according to the Bradford method [105]. The purity of the enzyme was analyzed by SDS-PAGE [106]. Fractions with pure *Se*LipC were pooled, and isopropanol was removed by dialysis against 15 mM Tris/HCl buffer pH 8.0 employing cellulose tubing from Medicell Membranes Ltd. (molecular weight cut-off of 12–14 kDa).

### 4.5. Protein Sequence and Structural Analysis of SeLipC

The N-terminal amino sequence of pure *Se*LipC was determined according to what was described [107]. After SDS-PAGE, the protein band was transferred to a PVDF membrane *Immobilon-P* (Millipore, Burlington, MA, USA) and sequenced by automatic Edman degradation in a Procise 494 Protein Sequencing System (Applied Biosystems Inc., Waltham, MA, USA) at the Protein Chemistry Facility of CIB-CSIC. The molecular mass and peptide mass fingerprinting analysis by MALDI-TOF/TOF mass spectrometry were performed using single and double fragmentation of given peptides (MS/MS) in the AB Sciex 4800 Plus MALDI TOF/TOF™ analyzer (Framingham, MA, USA) at the Proteomics Facility of UCM. Peptides were matched with MASCOT against the putative amino acid sequence of *Se*LipC and the SwissProt database with a taxonomic restriction to bacteria. Fluorescence spectroscopy studies of 0.035 mg/mL of pure lipase solution in 5 mM Tris/HCl buffer pH 8.0 were carried out in an Thermo Spectronic AMINCO-Bowman Series 2 spectrofluorometer (Madison, WI, USA) with a 1 cm pathlength cell at 25 °C. Fluorescence emission spectra were recorded from 295 to 450 nm, using 295 nm as the excitation wavelength. The excitation energy used was 875 V at 4 nm slit width and 8 nm for emission. Circular dichroism (CD) spectra of 0.29 mg/mL of pure enzyme solution in 5 mM Tris/HCl buffer pH 8.0 were recorded using a 0.1 cm pathlength quartz cuvette in the far-UV region from 190 to 260 nm using a Jasco J-715 spectropolarimeter (Japan Spectroscopic Company, Tokyo, Japan) equipped with a thermostated cell holder connected to a Neslab RTE-111 water bath (Thermo Scientific, Waltham, MA, USA). Thermal unfolding of pure lipase was studied by CD variation at 208 nm from 25 to 80 °C at a scan speed of 20 °C/h. All CD readings were expressed as the mean residue molar ellipticity (deg. cm^2^. dmol^−1^), and secondary structure information was obtained from CD spectra using the DichroWeb analysis website [108] and choosing the CDSSTR method with the reference set 7 [109]. The denaturation temperature (*Tm*) of pure enzyme was also determined by calorimetry. An aliquot of 0.8 mg/mL of pure *Se*LipC in 5 mM Tris/HCl buffer pH 8.0 was scanned by increasing the temperature from 15 °C to 70 °C at a heating rate of 30 °C/h and then cooled to 15 °C for nine cycles using a VP-DSC microcalorimeter (MicroCal, Northampton, MA, USA). Experimental results were analyzed by OriginPro 2021 software (OriginLab, Northampton, MA, USA) to determine the *Tm* and enthalpy of the unfolding process.

### 4.6. Standard Activity Assay Using p-Nitrophenyl Palmitate as Substrate (pNPP Assay)

Lipase activity was routinely determined using *p*-nitrophenyl palmitate (pNPP) as a substrate. A solution of 4 mM pNPP was freshly prepared in isopropanol. This substrate stock solution (solution B) was sonicated (100 W, 40 kHz) for 1 min in an ultrasonic bath (J.P. Selecta, Barcelona, Spain). Then, 20 µL of solution B was mixed with 160 µL of solution A (62.5 mM Tris/HCl pH 8.3, 0.125% (*w*/*v*) gum arabic, and 0.25% (*v*/*v*) sodium deoxycholate) and preincubated for 5 min at 37 °C before adding 20 µL of lipase solution (0.015 µg of protein). After incubation at 37 °C for 10 min, the enzymatic reaction was stopped by the addition of 100 µL of a 0.4 M Na_2_CO_3_ solution. This final mixture was centrifuged at 12,000 rpm. After centrifugation of the samples at 9000× *g* for 10 min at 4 °C, the released *p*-nitrophenol (pNP) was determined by measuring the absorbance at 405 nm in a Heales MB-580 microplate reader (Shenzhen, China). An appropriate control without enzyme was used to subtract the absorbance produced by the spontaneous hydrolysis of pNPP. All measurements were carried out in triplicate. One international activity unit (IU) was defined as the amount of enzyme producing 1 μmol of pNP per minute under the assay conditions.

### 4.7. Esterase Activity Assay Using Methyl Phenoxyacetate as Substrate (MPOA Assay)

Esterase assays were carried out using MPOA as substrate in 12-multiwell plates (Thermo Fisher Scientific, USA). A total of 80 μL of 500 mM MPOA solution in dimethyl sulfoxide (DMSO) was mixed with 1.62 mL of 5 mM sodium phosphate buffer pH 7.0 and preincubated for 5 min at 37 °C before adding 300 µL of lipase solution (3 µg of protein). This final mixture was incubated at 37 °C under 400 rpm orbital shaking using a ThermoShaker (ELMI SkyLine DTS-4). At different incubation times, aliquots of 20 µL of the enzymatic reaction were mixed with 20 µL of cold methanol and analyzed by HPLC using Agilent 1100 equipment (Santa Clara, CA, USA) and a Phenomenex (Torrance, CA, USA) Luna column C18(2), 250 × 4.6 mm (5 µM particle size), with a 65% (*v*/*v*) methanol solution containing 0.05% (*v*/*v*) phosphoric acid as mobile phase. The flow rate was fixed at 0.8 mL/min, and the UV detector was set at 260 nm. The retention times of product (POA) and substrate (MPOA) were 6.4 min and 8.0 min, respectively. An appropriate control without enzyme was used to determine the spontaneous hydrolysis of MPOA under the same assay conditions. All determinations were carried out in triplicate. One international activity unit (IU) was defined as the amount of enzyme producing 1 μmol of POA per minute under the assay conditions.

### 4.8. Rhodamine Plate Assay for Lipase Activity Detection

For this qualitative assay, a stock solution of rhodamine 6G (1 mg/mL) was prepared in distilled water and subsequently sterilized by filtration. The plate was prepared in two layers: the bottom one contained 2% (*w*/*v*) sterile agar in 50 mM Tris/HCl buffer pH 8.3, whereas the topcoat medium was made with cooled 2% (*w*/*v*) agar with 2.5% (*v*/*v*) olive oil and 0.001% (*w*/*v*) rhodamine 6G. Before pouring, the top layer was vigorously stirred and emulsified by sonication (100 W, 40 kHz) for 1 min in a Selecta Ultrasonic bath. Lipase samples (40 μL at 0.043 mg/mL) were loaded in 5-mm diameter wells aseptically made in the agar plate and incubated at 37 °C for 24 h. Results were analyzed by observing fluorescence halos surrounding the wells when irradiating at 302 nm in a transilluminator, UVIPro V1.0 (UVItec Limited, Cambridge, UK). 40 μL of lipase TL (Meito Sangyo Co., Nagoya, Japan) at 0.5 mg/mL and 40 μL of 15 mM Tris/HCl buffer pH 8.0 were used as positive and negative controls, respectively.

### 4.9. Detection of Hydrolytic Activity on Polyester-Type Plastics

A total of 0.5% PBSA, PBS, or PCL emulsions were prepared according to the procedure reported previously [110]: 2 g polymer pellet was dissolved in 40 mL of dichloromethane, followed by the addition of 100 mL of distilled water and 2 mL of a 2% (*w*/*v*) Sarkosil NL solution. After sonication of the mixture for 10 min using a Branson 250 probe, dichloromethane was evaporated by stirring at 80 °C for 2 h in a draft chamber. The emulsion was filled up to 400 mL with distilled water, and the pH was adjusted to 7.0. Next, mineral minimum [MM] agar plates containing 0.1% emulsified polymers were prepared as described [110]. Wells were aseptically cut in the agar plates, and 40 μL of lipase solution (0.043 mg/mL) were loaded into each well, whereas 15 mM Tris/HCl buffer pH 8.0 was loaded as the negative control. The plates were incubated for up to 72 h at 37 °C, and a visible, clear zone surrounding the well confirmed the enzymatic hydrolysis of the polyester-type plastics.

### 4.10. Biochemical Characterization of SeLipC

The optimal reaction conditions for *Se*LipC activity were determined using pNPP as a substrate by the standard assay described above. All experiments were carried out in triplicate. The optimum pH of pure *Se*LipC was established by assaying the lipase activity at 37 °C using different buffer systems: 50 mM MES (pH 5.0–7.0), 50 mM HEPES (pH 7–7.5), 50 mM Tris/HCl (pH 7.5–9.0), and 50 mM glycine/NaOH (pH 9.0–10.0). To determine pH stability, 20 µL of enzyme solution containing 0.015 µg of pure lipase was incubated in 5 mM of each buffer at different pH values for 10 min at 4 °C. Afterwards, residual lipase activity was measured using the standard pNPP assay. The optimum temperature was also determined by assaying the lipase activity at several temperatures (20–80 °C) in 15 mM Tris/HCl pH 8.0 buffer. Thermal stability studies were performed by incubating purified enzyme at either 0.72 µg/mL or 19 µg/mL in 15 mM Tris/HCl pH 8.0 for 10 min at different temperatures (20 to 80 °C) in a thermostatized water bath. After 10 min in the ice bath, residual activity was measured by a standard pNPP assay in the case of the lower enzyme concentration, whereas the MPOA assay was employed for the higher enzyme concentration. Furthermore, thermal inactivation studies of *Se*LipC were carried out by incubating 0.72 µg/mL or 19 µg/mL enzyme solution at 45 °C at different times, and the residual activity was assessed using pNPP or MPOA as substrate, respectively. Experimental plots of residual activity versus storage time were fitted to a single exponential decay using the SigmaPlot 11.0 program. The analysis of the experimental thermal deactivation data was carried out using a first-order kinetic model that allowed the calculation of the deactivation rate constant (*k_d_*) from the slope of the semilogarithmic plot, and the half-life (*t_1/2_* = ln 2/*k_d_*) was considered as the time required to reduce 50% of the initial enzyme activity. The effect of ionic strength (*I*) was studied by measuring the activity in the presence of different concentrations of NaCl up to 750 mM with the standard assay. Similarly, lipase activity of pure *Se*LipC was assayed in the presence of CuCl_2_, CaCl_2_, BaCl_2_, CoCl_2_, ZnCl_2_, MgCl_2_, FeCl_3_, FeSO_4_, and MnCl_2_ at 1 mM final concentration. The effect of water-miscible organic solvents (methanol, ethanol, isopropanol, DMSO, acetone, 1,4-dioxane, THF, DMF, and pyridine) on enzyme activity was also studied. For that purpose, pNPP was dissolved in each solvent, reaching a final concentration in the assay mixture that ranged from 10 to 40% (*v*/*v*). Likewise, the lipase activity of *Se*LipC was assessed in the presence of 0.2% (*w*/*v*) of several surfactants (sodium deoxycholate, SDS, CTAB, Tween 20, Triton X-100, and Tween 80). Determination of kinetic parameters for pNPP as substrate was performed under optimal assay conditions. Standard assays with increasing concentrations of pNPP ranging from 10 to 800 µM were carried out in 50 mM Tris/HCl buffer, pH 8.0, and 37 °C. Kinetics curves were fitted to the equation: *v* = (*V_max_* × S)/(*K_M_* + S), and *K_M_* and *k_ca_*_t_ were determined by nonlinear hyperbolic regression using the SigmaPlot 11.0 program (Systat Software Inc., Palo Alto, CA, USA). Finally, enzymatic assays were performed employing several *p*-nitrophenyl alkanoate esters as substrates: pNP-acetate (C2), pNP-butyrate (C4), pNP-valerate (C5), pNP-hexanoate (C6), pNP-octanoate (C8), pNP-decanoate (C10), pNP-laurate (C12), pNP-myristate (C14), pNP-palmitate (C16), and pNP-stearate (C18). All of them were dissolved in isopropanol at a final concentration of 4 mM and then employed in the same conditions as the described standard pNPP assay. Finally, the inhibition of *Se*LipC by phenylmethanesulfonyl fluoride (PMSF), a serine-specific modification reagent, was also addressed. For that purpose, 20 μL of enzyme solution (1.5 μg of SeLipC) were incubated with PMSF (1 mM and 10 mM in DMSO) for 30 min at 4 °C in 100 µL of final volume in Tris-HCl 15 mM, pH 7.0. The remaining enzyme activity was determined using the standard assay. In addition, to analyze protection by substrate against enzyme inactivation by PMSF, 20 μL of enzyme solution (1.5 μg of *Se*LipC) was preincubated with 10 μL of pNPP 4 mM for 5 min at 4 °C. Then, PMSF was added at a final concentration of 1 mM and 10 mM, and the mixture was incubated for 30 min at 4 °C in the corresponding buffer. After chemical modification, residual activity was assayed using the standard assay. Controls of the enzyme without PFMS were carried out in all the experiments.

### 4.11. Enzymatic Synthesis of Sugar Fatty Acid Esters

Different sugars were dried in a desiccator under vacuum for 24 h before their use, while *Se*LipC was dried by lyophilization. The THF solvent was distilled before being employed. Sugar substrates were dissolved in anhydrous THF (30 nM) in the presence of activated 3 Å molecular sieves (20 mg/mL). When the substrate was not completely soluble in THF, the mixture was sonicated for 10 min before starting the enzymatic reaction. *Se*LipC was added to the medium (4 IU/mL, pNPP assay), and the reaction was started by the addition of vinyl laurate (3 equiv). The reaction was incubated at 35 °C with orbital shaking (200 rpm), and samples were withdrawn at different reaction times and then filtered through a 0.22 µm PTFE (polytetrafluoroethylene) syringe filter before HPLC analysis. Such analysis was performed using a Jasco HPLC equipped with a Mediterranean Sea 18 column (15 × 0.46 × 5 mm) (Teknokroma Analítica S.A., Barcelona, Spain), employing acetonitrile:H_2_O 70:30 (*v*/*v*) as the mobile phase. The flow rate was fixed at 0.7 mL/min, and an evaporative light scattering detector (Sedex LT-ELSD-80LT) was used to analyze the monoesterified products synthetized by *Se*LipC.

## 5. Conclusions

In this work, in silico searching allowed us to find four putative lipase-encoding genes in the *Streptomyces exfoliatus* DMSZ 41693 genome. Two lipases (abbreviated as *Se*LipA and *Se*LipC) were successfully cloned and extracellularly expressed in *Rhodococcus* sp. T104 KACC 21099 as hosts. After its purification by hydrophobic chromatography, the *Se*LipC structure was studied by circular dichroism (CD), differential scanning calorimetry (DSC), and fluorescence. In addition, the lipase was kinetically characterized using *p*-nitrophenyl palmitate (pNPP) as a substrate. In this sense, kinetic parameters for pNPP, the effect of pH and temperature on enzyme activity and stability, and the effect of metal ions, detergents, and water-miscible organic solvents were exhaustively studied. Likewise, the effect of reducing agents on lipase activity was examined. The results suggest that those cysteine residues, suspected to be involved in disulfide bonds, are not needed for activity. In addition, the chemical modification studies of *Se*LipC with PMSF indicate that a serine residue is involved at the active site of the enzyme. To complete this characterization, a novel esterase assay for lipase activity determination was established using methyl phenoxyacetate (MPOA) as a substrate. Likewise, the hydrolytic activity of *Se*LipC towards olive oil and several polyester-type plastics such as poly(butylene succinate), poly(butylene succinate)-*co*-(butylene adipate), and poly(ε-caprolactone) was confirmed. Finally, *Se*LipC can catalyze the enzymatic synthesis of different sugar esters from Glc, Rha, Fuc, GlcNAc, and GalNAc, although further research is needed to improve reaction yields. In conclusion, the biotechnological application of *Se*LipC should be seriously considered.

## Data Availability

The data presented in this study are fully available in the main text and Supplementary Material of this article.

## Supplementary Materials

The following supporting information can be downloaded at: https://www.mdpi.com/article/10.3390/ijms242317071/s1.
