# Supplementary material for: A Novel Lipase from Streptomyces exfoliatus DSMZ 41693 for Biotechnological Applications
Source: Int J Mol Sci. 2023 Dec 2;24(23):17071. doi: 10.3390/ijms242317071 (PMC10707221; doi:10.3390/ijms242317071)
Supplement: Supplementary file 1 [file ijms-24-17071-s001.zip › ijms-2693348-supplementary.pdf]

Supplementary material

A novel lipase from *Streptomyces exfoliatus* DSMZ 41693 for biotechnological applications

Guillermo Rodríguez-Alonso[1 #], Juan Toledo-Marcos[1 † #], Lara Serrano-Aguirre[1 ‡], Carlos Rumayor[1], Beatriz Pasero[1 §], Aida Flores[2], Ana Saborido[1], Pilar Hoyos[2], María J. Hernáiz[2], Isabel de la Mata[1 *], Miguel Arroyo[1 *]

[1] Department of Biochemistry and Molecular Biology, Faculty of Biology, Universidad Complutense de Madrid (UCM), E-28040 Madrid (Spain); guillr05@ucm.es (G.R.-A.); juan.toledo-marcos@medunigraz.at (J. T.-M.); lara.serrano@cib.csic.es (L.S.-A.); carlorum@ucm.es (C.R.); beatriz.pasero@cib.csic.es (B.P.); asaborid@ucm.es (A.S.).

[2] Department of Chemistry in Pharmaceutical Sciences, Faculty of Pharmacy, Universidad Complutense de Madrid (UCM), E-28040 Madrid (Spain); aiflores@ucm.es (A.F.); phoyosvi@ucm.es (P.H.); mjhernai@ucm.es (M.J.H.).

† Present address: Gottfried Schatz Research Center, Medical Physics and Biophysics, Medical University of Graz, Neue Stiftingtalstraße 6/III, A-8010 Graz (Austria).

‡ Present address: Polymer Biotechnology Group, Department of Microbial & Plant Biotechnology, Center for Biological Research (CIB), Spanish National Research Council (CSIC), c/ Ramiro de Maeztu 9, E-28040 Madrid (Spain).

§ Present address: Environmental Biotechnology Group, Department of Microbial & Plant Biotechnology, Center for Biological Research (CIB), Spanish National Research Council (CSIC). c/ Ramiro de Maeztu 9, E-28040 Madrid (Spain).

# These authors contributed equally to this work.

* Correspondence: idlmata@ucm.es (I.d.l.M.); marroyos@ucm.es (M.A.). Tel.: +34-913944150. These two researchers share the position of last author.

Table S1. Strains, plasmids, and oligonucleotides used in this study.

**Strain**	**Relevant description or sequence**	**Reference**
Streptomyces exfoliatus DSMZ 41693	Producer of lipases	[26]
Escherichia coli DH5α	Host for recombinant plasmids<br>F^{-} ϕ80*lac*ZΔM15 Δ(*lac*ZYA-*arg*F)U169 *rec*A1 *end*A1 *hsd*R17(r_K^-, m_K^+) *pho*A *sup*E44 λ^- *thi*-1 *gyr*A96 *rel*A1	[101]
Rhodococcus sp. T104 KACC 21099	Host for gene expression (Kn^s)	[102]
Rhodococcus sp. T104 KACC 21099 pENV19*Se*LipA	*Rhodococcus sp.* T104 KACC 21099 recombinant strain harboring the plasmid pENV19*Se*lipA	This study
Rhodococcus sp. T104 KACC 21099 pENV19*Se*LipB	*Rhodococcus sp.* T104 KACC 21099 recombinant strain harboring the plasmid pENV19*Se*lipB	This study
Rhodococcus sp. T104 KACC 21099 pENV19*Se*LipC	*Rhodococcus sp.* T104 KACC 21099 recombinant strain harboring the plasmid pENV19*Se*lipC	This study
Rhodococcus sp. T104 KACC 21099 pENV19*Se*LipD	*Rhodococcus sp.* T104 KACC 21099 recombinant strain harboring the plasmid pENV19*Se*lipD	This study
**Plasmids**		
pENV19	Shuttle vector for *E. coli-Rhodococcus* with the constitutive p*ermE** promoter (5.1 Kb, Kn^R pAL5000ori p*ermE** ColE1ori)	[35,37,38]
pENV19*Se*LipA	pENV19 containing *lipA* gene from *S. exfoliatus* DSMZ 41693	This study
pENV19*Se*LipB	pENV19 containing *lipB* gene from *S. exfoliatus* DSMZ 41693	This study
pENV19*Se*LipC	pENV19 containing *lipC* gene from *S. exfoliatus* DSMZ 41693	This study
pENV19*Se*LipD	pENV19 containing *lipPs* gene from *S. exfoliatus* DSMZ 41693	This study
**Primers**	Sequence (5’→3’)[#]	
*Se*LipAFWsp	GC**TCTAGA**GA*GGACG*ACTCCGTCATGAAACGACAC	This study
*Se*LipAREV	G**GAATTC**TCAGGAACCGAGCGGGCAGGTG	This study
*Se*LipBFWsp	GC**TCTAGA***GGAGG*TTCCATGAGACTGTCC	This study
*Se*LipBREV	G**GAATTC**CTACGCCGCCCCGTTG	This study
*Se*LipCFWsp	GC**TCTAGA**CAGTCGTGGCT*GGAACG*ACTGGTCCTGCAAGCCC	This study
*Se*LipCREV	G**GAATTC**CCTAGCCGATCACCGAGAGGCAGGTGGTCGGGGTCG	This study
*Se*LipDFW	GC**TCTAGA**A*GGAGT*TCCTATGTCAGGGAG	This study
*Se*LipDREV	GG**AATTC**TCAGGCCGTGGGACCGGGAATCTG	This study

[#]The restriction sites *Xba*I and *EcoR*I are shown in bold, ribosomal binding site sequence is shown in italics, and the start and the stop codons are shown underlined.

Table S2. PCR conditions for amplification of lipase genes from *Streptomyces exfoliatus* DSMZ 41693.

**Step**	**Temperature (ºC)**	**Time**
1. Denaturalization	95	3 minutes
2. Denaturalization	95	30 seconds
3. Annealing	83	30 seconds
4. Extension	72	55 seconds
5. Repeat 17 cycles steps 2-4 decreasing 1 °C annealing temperature by cycle		
6. Denaturalization	95	30 seconds
7. Annealing	58-72	30 seconds
8. Extension	72	55 seconds
9. Repeat 20 cycles steps 6-8 increasing 1 s annealing time by cycle		
10. Extension	72	10 minutes

Table S3. *p*NP-ester hydrolytic specific activity in the culture broth of the recombinant strains**.**

pNP-esters (number of carbon atoms in acyl chain)	Specific activity (IU/mg protein)			
	***Se*LipA**	***Se*LipB**	***Se*LipC**	***Se*LipD**
pNP-acetate (C2)	n.d.	n.d.	n.d.	n.d.
pNP-butyrate (C4)	0.9	n.d.	0.9	n.d.
pNP-valerate (C5)	2.5	n.d.	2.5	n.d.
pNP-hexanoate (C6)	3.1	n.d.	6.5	n.d.
pNP-octanoate (C8)	4.8	n.d.	13.3	n.d.
pNP-decanoate (C10)	8.0	n.d.	17.2	n.d.
pNP-laurate (C12)	7.1	n.d.	12.8	n.d.
pNP-myristate (C14)	4.8	n.d.	9.5	n.d.
pNP-palmitate (C16)	5.3	n.d.	12	n.d.
pNP-stearate (C18)	4.6	n.d.	7.8	n.d.

n.d.: not detected

Table S4. Purification of *Se*LipC produced by *Rhodococcus* pENV19*Se*LipC by hydrophobic interaction chromatography (HIC).

Step	V (mL)	Activity (IU)	Protein concentration (mg/mL)	Total protein (mg)	Specific activity (IU/mg)	Purification factor (fold)	Yield (%)
**Broth**	700	1356	0.163	114.1	12	1	100
**Broth $(NH_4)_2SO_4$**	740	1202	0.151	111.7	11	0.9	89
**HIC**	92	245.7	0.023	2.12	116.6	3.6	17.2
**Dialysis**	140	240.8	0.007	1.04	232.4	7.2	16.9

Table S5. Effect of different reducing agents on *Se*LipC activity.

Chemical reagent	Concentration	Residual activity (%)
No addition	-	100.0 ± 7.2
Dithiothreitol (DTT)	1 mM	94.9 ± 6.0
	10 mM	101.6 ± 5.7
2-Mercaptoethanol	1 mM	99.8 ± 1.9
	10 mM	99.4 ± 2.9

Table S6. Effect of chemical modification with PMSF on *Se*LipC.

Chemical reagent	Concentration	Residual activity (%)
In absence of substrate (active site not protected)		
No addition	-	100 ± 2.6
PMSF	1 mM	84.7 ± 3.5
	10 mM	51.2 ± 5.5
In presence of substrate (active site protected)		
No addition	-	100 ± 0.5
PMSF	1 mM	100.5 ± 1.2
	10 mM	90.0 ± 1.3

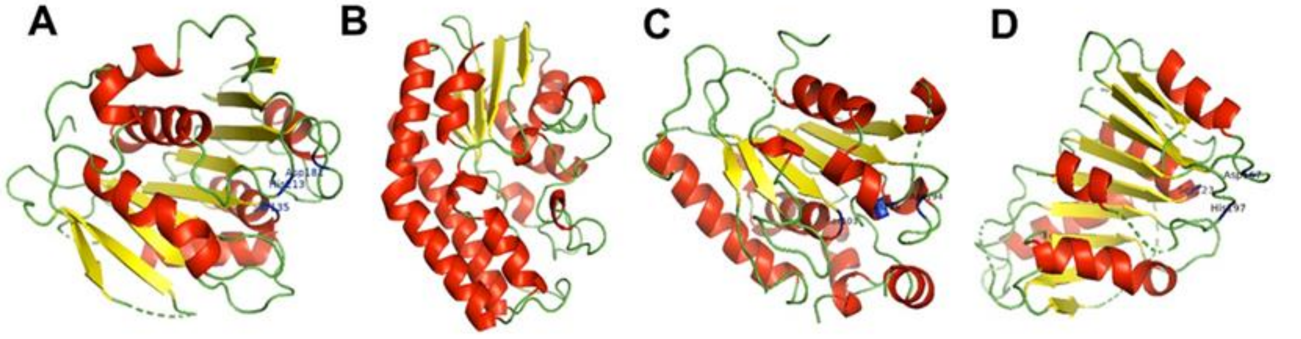

Figure S1. Predicted 3D structures of hypothetical lipases from *Streptomyces exfoliatus* DSMZ 41693. **A.** *Se*LipA; **B.** *Se*LipB; **C.** *Se*LipC; **D.** *Se*LipD. Models were obtained by RoseTTAFold program [29] and their structures were visualized by PyMOL program (Schrodinger, LLC, version 2.5).

gi|639917083 Mass: 31194 Score: 659 Expect: 1.3e-066 Matches: 21
lipase [*Streptomyces exfoliatus*]

Query	Start	–	End	Observed	Mr(expt)	Mr(calc)	ppm	M	Score	Peptide
59	84	–	109	2896.5251	2895.5178	2895.4418	26.2	0		R.GYCVFSLDYGQLPNVPFFHGLGPIAK.S
5	110	–	120	1266.5919	1265.5846	1265.5888	-3.28	0		K.SAEQLDAYVDR.V
6	110	–	120	1266.5919	1265.5846	1265.5888	-3.28	0	66	K.SAEQLDAYVDR.V
72	110	–	143	3544.8044	3543.7971	3543.6722	35.2	1		K.SAEQLDAYVDRVLAATGAPEADLVGHSQGGMMPR.W + 2 Oxidation (M)
39	121	–	143	2297.1086	2296.1013	2296.0940	3.19	0		R.VLAATGAPEADLVGHSQGGMMPR.W + 2 Oxidation (M)
56	121	–	147	2887.5613	2886.5540	2886.4157	47.9	1		R.VLAATGAPEADLVGHSQGGMMPRWYLK.F + 2 Oxidation (M)
48	148	–	175	2836.6228	2835.6155	2835.5495	23.3	1		K.FLGGAEKVNTLVGIAPDNHGTTLLGLTK.L
32	155	–	175	2134.1792	2133.1719	2133.1794	-3.50	0		K.VNTLVGIAPDNHGTTLLGLTK.L
33	155	–	175	2134.1792	2133.1719	2133.1794	-3.50	0	69	K.VNTLVGIAPDNHGTTLLGLTK.L
67	176	–	206	3163.7524	3162.7451	3162.6489	30.4	0		K.LLPYFPGAEDLISSATPGLADQIAGSAFITK.L
68	176	–	206	3163.7524	3162.7451	3162.6489	30.4	0	112	K.LLPYFPGAEDLISSATPGLADQIAGSAFITK.L
3	207	–	218	1213.6143	1212.6070	1212.6099	-2.35	0		K.LNEGGDTVPGVR.Y
4	207	–	218	1213.6143	1212.6070	1212.6099	-2.35	0	42	K.LNEGGDTVPGVR.Y
27	219	–	234	1939.9414	1938.9341	1938.9476	-6.94	0		R.YHVIASQYDEVVTPYR.S
28	219	–	234	1939.9414	1938.9341	1938.9476	-6.94	0	143	R.YHVIASQYDEVVTPYR.S
29	219	–	234	1939.9414	1938.9341	1938.9476	-6.94	0	152	R.YHVIASQYDEVVTPYR.S
22	235	–	251	1859.9799	1858.9726	1858.9789	-3.37	0		R.SQFLSGPNVTNVLIQDK.C
12	252	–	267	1755.8586	1754.8513	1754.8621	-6.16	0		K.CALDLSEHVAIGTVDR.V
70	252	–	281	3276.6643	3275.6570	3275.6357	6.51	1		K.CALDLSEHVAIGTVDRVTFHEVANALDPAR.A
9	268	–	281	1539.7820	1538.7747	1538.7841	-6.12	0		R.VTFHEVANALDPAR.A
10	268	–	281	1539.7820	1538.7747	1538.7841	-6.12	0	70	R.VTFHEVANALDPAR.A

Figure S2. Peptide mass fingerprinting of *Se*LipC obtained by MALDI-TOF analysis. The protein was identified as a lipase from *Streptomyces exfoliatus* (gi|639917083) after peptide matching process using MASCOT Software (Score 659).

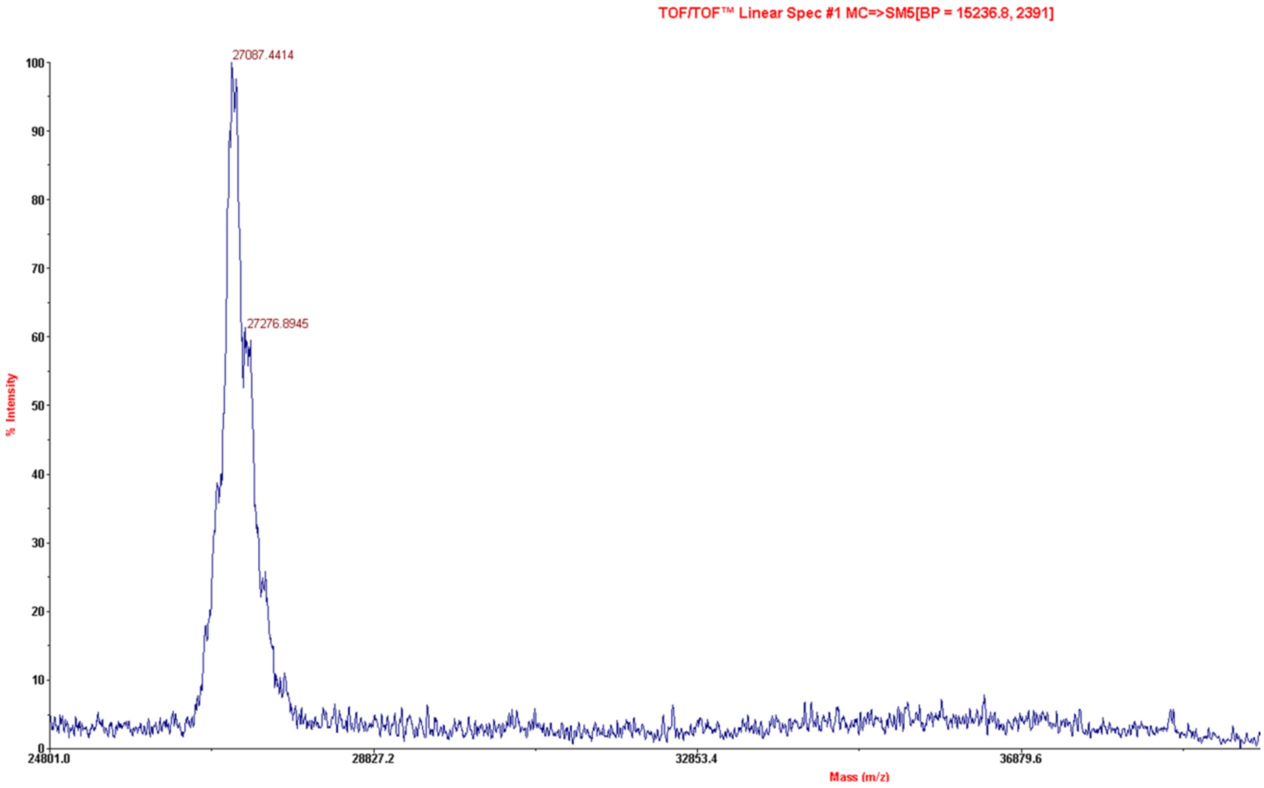

Figure S3. Determination of the molecular mass of *Se*LipC by MALDI-TOF. Mass spectrum recorded with 0.127 mg/mL of pure lipase.

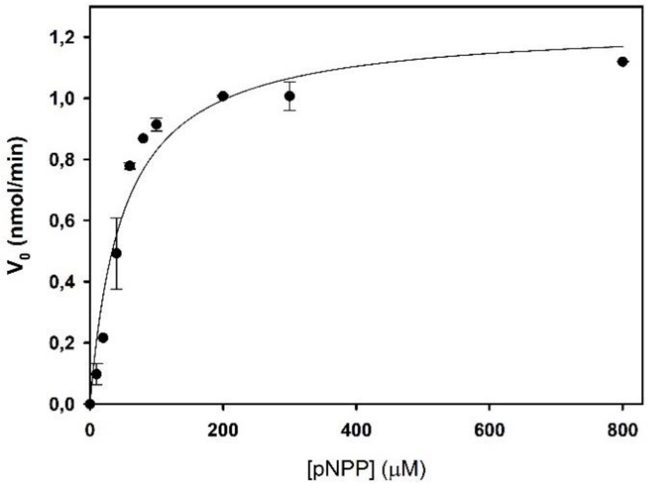

Figure S4. Michaelis-Menten hyperbola plot for the determination of kinetic parameters of *Se*LipC using pNPP as substrate. Conditions: 200 µL assay volume, 0.05 µg/mL of enzyme (1.81 nM), assay time 10 min, 37°C, 50 mM Tris HCl pH 8.0, 0.01% gum arabic and 0.02% sodium deoxycholate. K_M = 49.6 ± 11.2 µM and V_{max} = 1.24 ± 0.08 × 10^{-3} µmoles/min (k_{cat} = 57 s^{-1} and k_{cat} / K_M = 1.15 × 10^6 s^{-1}·M^{-1}).

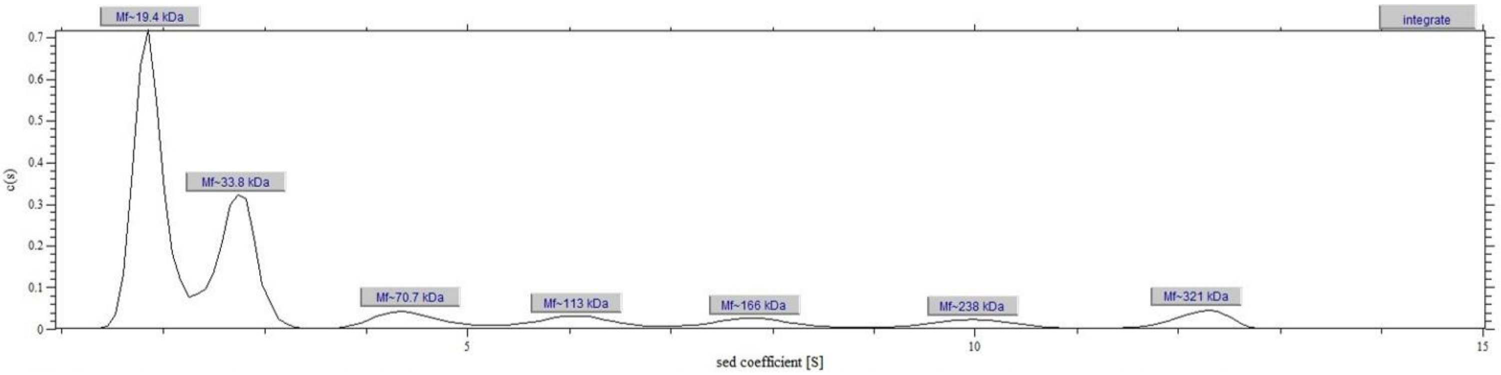

Figure S5. Analytical ultracentrifugation analysis of *Se*LipC: c(s) distribution as a function of sedimentation coefficient (*S*). Experiment was performed with 0.320 mg/mL of pure lipase, and the presence of two main species with sedimentation coefficient (*S*) values of 1.9 and 2.7 were observed. These values are compatible with the presence of monomers and dimers with spheroid oblate-shape in both forms.
